# Effect of Probiotic Fermented Milk Supplementation on Glucose and Lipid Metabolism Parameters and Inflammatory Markers in Patients with Type 2 Diabetes Mellitus: A Meta-Analysis of Randomized Controlled Trials

**DOI:** 10.3390/biology13080641

**Published:** 2024-08-21

**Authors:** Hao Zhong, Lingmiao Wang, Fuhuai Jia, Yongqiu Yan, Feifei Xiong, Khemayanto Hidayat, Yunhong Li

**Affiliations:** 1 School of Medicine, Nankai University, Tianjin 310071, China; zhonghao@zjut.edu.cn; 2 College of Food Science and Technology, Zhejiang University of Technology, Hangzhou 310014, China; 3 Ningbo Yufangtang Biotechnology Co., Ltd., Ningbo 315012, Chinaxiongfeifei1214@sina.com (F.X.); 4 Department of Nutrition and Food Hygiene, School of Public Health, Suzhou Medical College of Soochow University, Suzhou 215123, China

**Keywords:** probiotic, fermented milk, blood glucose, inflammation, meta-analysis

## Abstract

**Simple Summary:**

In the present meta-analysis of randomized controlled trials, probiotic fermented milk supplementation appeared to be beneficial in lowering the levels of fasting plasma glucose, HbA1c, total cholesterol, and C-reactive protein. While these findings are encouraging, they should be interpreted cautiously, as considerable limitations of the included trials and analyses precluded solid conclusions.

**Abstract:**

Modulating gut microbiota composition through probiotic administration has been proposed as a novel therapy for type 2 diabetes mellitus (T2DM), and fermented milk is arguably the most common and ideal probiotic carrier. The present meta-analysis was performed to assess the effects of probiotic fermented milk supplementation on glucose and lipid metabolism parameters and inflammatory markers in patients with T2DM using published data from randomized controlled trials (RCTs). The PubMed, Web of Science, and Cochrane Library databases were searched for relevant RCTs. A random-effects model was used to generate the weighted mean difference (WMD) and 95% confidence interval (95% CI). Probiotic fermented milk supplementation reduced the levels of fasting plasma glucose (MD = −17.01, 95% CI −26.43, −7.58 mg/dL; n = 7), hemoglobin A1c (MD = −0.47, 95% CI −0.74, −0.21%; n = 7), total cholesterol (MD = −5.15, 95% CI −9.52, −0.78 mg/dL; n = 7), and C-reactive protein (MD = −0.25, 95% CI −0.43, −0.08; n = 3) but did not significantly affect the levels of HOMA-IR (MD = −0.89, 95% CI −2.55, 0.78; n = 3), triglyceride (MD = −4.69, 95% CI −14.67, 5.30 mg/dL; n = 6), low-density lipoprotein cholesterol (MD = −4.25, 95% CI −8.63, 0.13 mg/dL; n = 7), high-density lipoprotein cholesterol (MD = 1.20, 95% CI −0.96, 3.36 mg/dL; n = 7), and tumor necrosis factor-alpha (MD: −0.58, 95% CI −1.47, 0.32 pg/mL; n = 2). In summary, the present findings provide a crude indication of the potential benefits of probiotic fermented milk supplementation in improving glucose and lipid metabolism and inflammation in patients with T2DM. However, more robust evidence is needed to determine the clinical significance of probiotic fermented milk in the management of T2DM.

## 1. Introduction

Diabetes mellitus (DM) is a metabolic disorder characterized by chronic hyperglycemia resulting from defects in insulin secretion, insulin action, or a combination of both. If left uncontrolled, the chronic hyperglycemia of diabetes can lead to long-term complications relating to different organs, particularly the eyes, blood vessels, heart, kidneys, and nerves [1]. According to the International Diabetes Federation, just over half a billion adults aged 20–79 were diagnosed with DM (the vast majority are type 2 diabetes mellitus [T2DM]) in 2021, accounting for 10.5% of the world’s adult population, and this number is projected to rise by nearly a quarter billion in 2045 [2,3]. Therefore, it is important to find effective interventions that can prevent DM or delay its progression to complications.

In T2DM, chronic hyperglycemia manifests when insulin secretion fails to compensate for resistance to insulin action [1]. Apart from the irregular metabolism of glucose, individuals with type 2 diabetes frequently exhibit dyslipidemia characterized by abnormal lipid profiles, such as decreased high-density lipoprotein cholesterol (HDL-C), increased low-density lipoprotein cholesterol (LDL-C), and triglycerides (TGs), which are the risk factors for cardiovascular disease [4,5]. Moreover, chronic low-grade inflammation is believed to play a crucial role in T2DM pathogenesis and is intensified in the presence of comorbid conditions (e.g., obesity, dyslipidemia, and hypertension) to promote the development of DM complications [6]. T2DM is largely preventable by implementing lifestyle modifications, such as adopting a healthy diet, maintaining a healthy body weight, being physically active, being sober, and not smoking. Lifestyle modifications also play a crucial role in managing T2DM and reducing its complications [7].

A growing body of research indicates that the composition of the gut microbiota may have a role in the onset of type 2 diabetes by influencing insulin sensitivity, intestinal permeability, energy balance, inflammatory regulation, and glycolipid metabolism [8]. Inflammation, insulin resistance, dyslipidemia, and hyperglycemia can result from gut microbiota dysbiosis, which can also contribute to increased intestinal permeability and the entry of bacterial endotoxins into the bloodstream. Furthermore, dysbiosis of the gut microbiota is one of the most important causal factors in the development of T2DM [9,10]. Modulating gut microbiota composition by administering adequate amounts of live beneficial microbes known as probiotics has been proposed as a potential therapy for T2DM.

Probiotics are commonly administered by consuming probiotic-containing supplements or foods. Dairy products, particularly fermented milk (e.g., yogurt and kefir), are some of the most common probiotic carriers [11]. Dairy products are often preferred as carriers over other foods as they contain properties that help the survival and growth of probiotics in the gut. The high buffering capacity and fat content of dairy products are believed to protect against harsh conditions in the guts, such as exposure to bile acid, gastric acid, and digestive enzymes, thereby improving the survival rates of probiotics [11,12,13,14]. Moreover, certain dairy-specific constituents have been suggested to promote the growth of probiotics [13].

A meta-analysis of randomized controlled trials (RCTs) found that probiotic fermented milk supplementation did not significantly affect glucose metabolism parameters (fasting plasma glucose [FPG], hemoglobin A1c [HbA1c], and homeostatic model assessment of insulin resistance [HOMA-IR]) in T2DM [15]. However, there are some caveats regarding the reliability and validity of their findings. The selection of weighted mean difference instead of standardized mean difference when continuous outcomes using different measurement scales can be easily converted seems inappropriate. Furthermore, the effects of probiotic fermented milk supplementation on lipid metabolism and inflammation, which are important factors in the progression of T2DM and its complications, have not been quantified. Given these considerations, a meta-analysis of RCTs was performed to provide up-to-date evidence on the effects of probiotic fermented milk on glucose and lipid metabolism parameters and inflammatory markers.

## 2. Materials and Methods

The present meta-analysis was performed and reported according to Preferred Reporting Items for Systematic Reviews and Meta-Analyses (PRISMA) guidelines [16]. The protocol for the present meta-analysis was pre-registered in PROSPERO (CRD42024537840). Two reviewers (HZ and LMW) independently performed the literature search, data extraction, study selection, and risk of bias assessment. Any disagreement was resolved via consensus.

### 2.1. Literature Search

The PubMed, Web of Science, and Cochrane Library databases were searched for relevant articles written in English up to 5 April 2024. The complete search strategy for the three databases is reported in Table S1. Furthermore, the references of relevant review articles and included trials were hand-searched to identify additional eligible studies.

### 2.2. Eligibility Criteria and Study Selection

The inclusion and exclusion criteria according to the Population, Intervention, Comparison, Outcomes, and Study (PICOS) framework are shown in Table 1. Briefly, RCTs that enrolled patients with T2DM were included if they investigated the effects of probiotic fermented milk supplementation on milk on the selected glucose (FPG, HbA1c HOMA-IR) and lipid (total cholesterol (TC), TG, LDL-C, and HDL-C) metabolism parameters and inflammatory markers (tumor necrosis factor-alpha (TNF-α) and C-reactive protein (CRP)).

### 2.3. Data Collection

The following data were extracted from the retrieved articles into a standardized form: trial authors, year of publication, the mean age of trial participants, countries where the trials were performed, number of participants, trial intervention, trial duration, and pre-and post-intervention values of the outcomes.

### 2.4. Assessment of Risk of Bias in Included Studies

The risk of bias in the included studies was assessed using the Cochrane Risk of Bias assessment tool [17]. This tool includes the assessment of the method of randomization, allocation concealment, performance bias, detection bias, attrition bias, reporting bias, and any other bias.

### 2.5. Statistical Analysis

The weighted mean difference (WMD) was employed as the summary measure of effect sizes. WMD was preferred due to variables being reported in different units of measure. Due to the methodological differences between interventions, a random-effects model was used to estimate the pooled effect sizes and 95% confidence intervals (CIs). The mean difference, standard deviation (SD), and sample size from each RCT are required to estimate the pooled effect sizes. If not available, the SD was calculated from the reported standard error (SE), confidence interval (CI), or *p*-value using the standard equation. For parallel RCTs, the mean difference was computed by subtracting the mean changes in glucose and lipid metabolism parameters and inflammatory markers from the baseline to the endpoint in the placebo group from those in the intervention group. For cross-over RCTs, the effect sizes were computed by subtracting the mean values of glucose and lipid metabolism parameters and inflammatory markers at the end of the placebo period from those reported at the end of the intervention period. Test for publication bias and subgroup analysis was not performed due to the limited RCTs in each analysis (n ≤ 9 RCTs, Figures S1–S4). The degree of heterogeneity across the included RCTs was evaluated using I^2^ statistics. The I^2^ values < 25%, 25–50%, and >50% indicated low, moderate, and high heterogeneity, respectively. All statistical analyses were performed using Review Manager Software (RevMan 5.3; Cochrane Collaboration, Oxford, England).

## 3. Results

### 3.1. Literature Search

**Figure 1:**
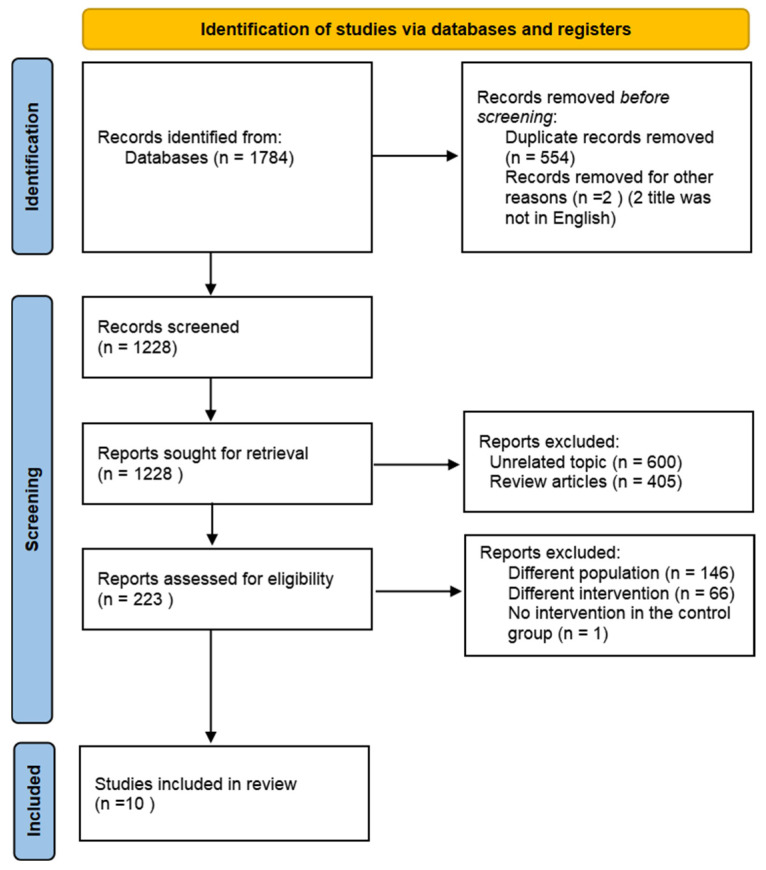
Flow diagram of the literature search procedure.

A comprehensive overview of the study selection process is presented in Figure 1. The electronic search across three main databases identified 1784 records. After excluding non-English articles and screening abstracts or titles, 223 articles were available for full-text screening. Of these articles, 213 articles were excluded, leaving 10 articles that met the inclusion criteria [18,19,20,21,22,23,24,25,26,27].

### 3.2. Characteristics of Included Trials

The characteristics of the included RCTs are summarized in Table 2. More than half of these RCTs were conducted in Iran [18,19,20,21,23,24,25,26], while the remaining were conducted in Denmark [22] and Brazil [27]. All included RCTs were parallel, double-blinded trials. The duration of the intervention ranged from 6 weeks to 16 weeks. Most trials used conventional fermented milk containing probiotics (mostly *Lactobacillus bulgaricus* and *Streptococcus thermophilus*) as the control for probiotic fermented milk. Probiotic fermented milk was conventional fermented milk enriched with additional probiotic strains (mostly Lactobacillus acidophilus La5 and *Bifidobacterium lactis* Bb12). The daily amount of fermented milk consumed ranged from 100 g/d to 600 g/d. The daily count of additional probiotics obtained from probiotic fermented milk ranged from 7.3 × 10^8^ cfu/day to 6.26 × 10^10^ cfu/d. One [22] trial used single-strain probiotics, one [19] did not disclose probiotic strain, and nine [18,20,21,23,24,25,26,27] used multi-strain probiotics. All RCTs asked the participants in the probiotic fermented milk and control groups to continue their dietary and lifestyle habits.

### 3.3. Risk of Bias

**Figure 2:**
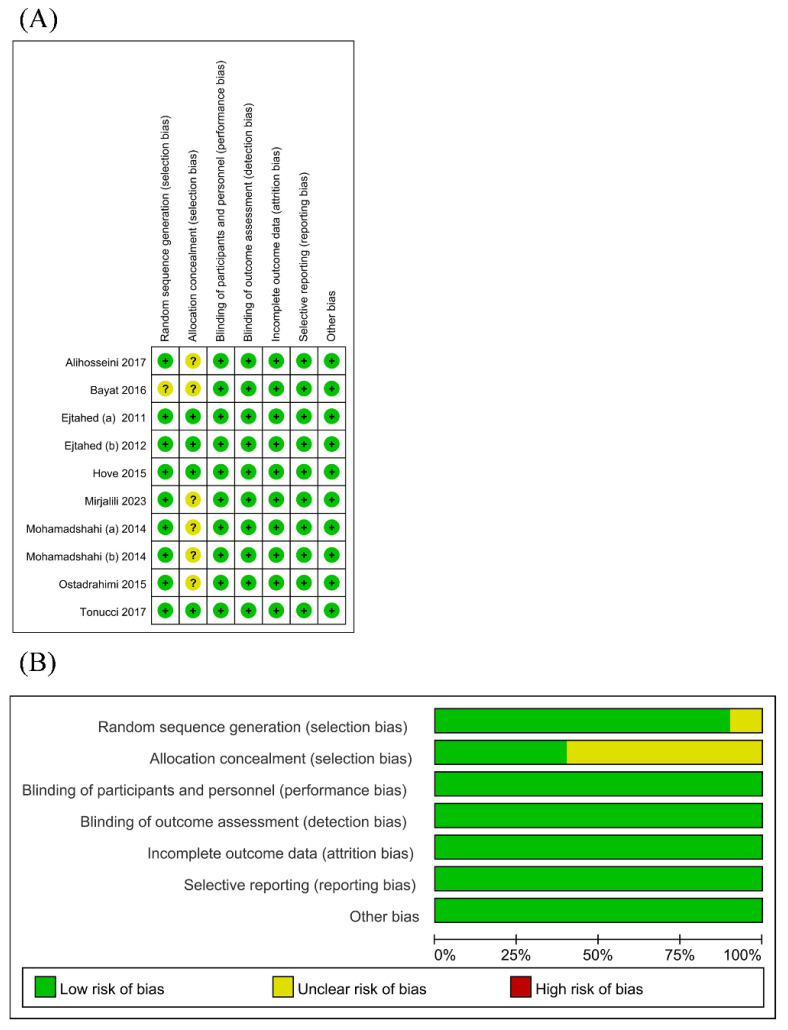
Risk of bias graph (**A**) [18,19,20,21,22,23,24,25,26,27] and risk of bias summary (**B**) for all RCT studies.

The risk of bias assessment is presented in Figure 2. Nearly all RCTs appropriately generated a random sequence. Only a few RCTs adequately ensured allocation concealment. Since the outcomes were based on objective measurements (i.e., glucose and lipid metabolism parameters and inflammatory markers), which were unlikely to be affected by the lack of blinding, the risks of performance and detection bias were deemed low in all RCTs. The risk of attrition bias was low as considerable loss to follow-up was not an issue in all RCTs. The risk of selective reporting was low as all RCTs provided accessible trial protocols.

### 3.4. Glucose Metabolism Parameters

**Figure 3:**
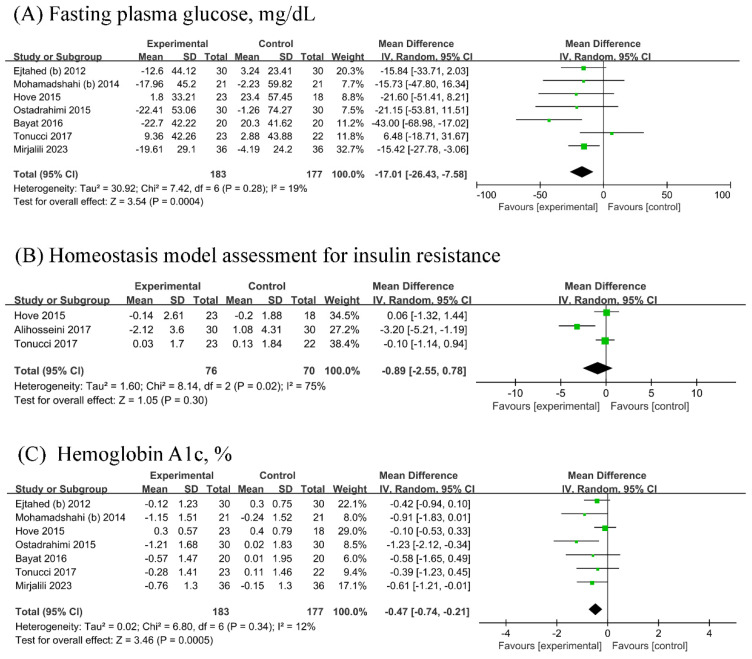
Forest plot for the effect of probiotic fermented milk on (**A**) fasting plasma glucose [19,21,22,23,25,26,27], (**B**) homeostasis model assessment for insulin resistance [18,22,27], and (**C**) hemoglobin A1c [19,21,22,23,25,26,27].

Seven RCTs (n probiotic fermented milk/n control = 183/177) each were included in the analyses of FPG and HbA1c, while three [18,22,27] RCTs were included in the analysis of HOMA-IR (n probiotic fermented milk/n control = 76/70). Probiotic fermented milk supplementation significantly reduced the levels of FPG (MD = −17.01, 95% CI −26.43, −7.58, *p* = 0.0004; Figure 3A) and HbA1c (MD = −0.47, 95% CI −0.74, −0.21, *p* = 0.0005; Figure 3C) but did not significantly affect HOMA-IR levels (MD = −0.89, 95% CI −2.55, 0.78, *p* = 0.30; Figure 3B). High heterogeneity was observed for HOMA-IR (I^2^ = 75%), whereas low heterogeneity was observed for other outcomes (All I^2^ ≤ 19%).

### 3.5. Lipid Metabolism Parameters

**Figure 4:**
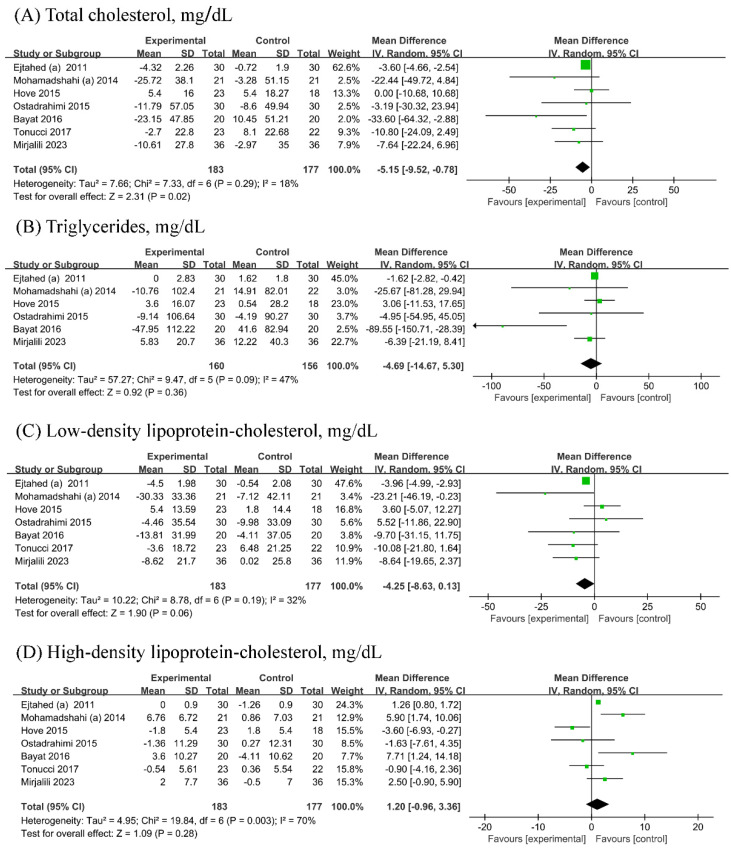
Forest plot for the effect of probiotic fermented milk on (**A**) total cholesterol [19,20,22,23,24,26,27], (**B**) triglycerides [19,20,22,23,24,26], (**C**) low-density lipoprotein-cholesterol [19,20,22,23,24,26,27], and (**D**) high-density lipoprotein-cholesterol [19,20,22,23,24,26,27].

Seven [19,21,22,23,24,26,27] RCTs (n probiotic fermented mlik/n control = 183/177) each were included in the analyses of TC, LDL-C, and HDL-C, while six [19,21,22,23,24,26] RCTs (n probiotic fermented milk/n control = 160/156) were included in the analyses of TG. Probiotic fermented milk supplementation reduced the levels of TC (−5.15, 95% CI −9.52, −0.78 mg/dL; Figure 4A) but did not significantly affect the levels of TG (−4.69, 95% CI −14.67 mg/dL; Figure 4B), LDL-C (MD = −4.25, 95% CI −8.63, 0.13 mg/dL; Figure 4C), and HDL-C (1.20, 95% CI −0.96, 3.36 mg/dL; Figure 4D). Low heterogeneity was observed for TC (I^2^ = 18%), whereas low-to-moderate heterogeneity was observed for other outcomes (All I^2^ ≥ 32%).

### 3.6. Inflammatory Markers

**Figure 5:**
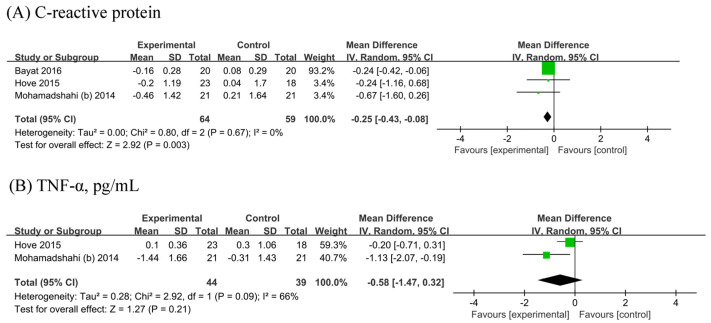
Forest plot for the effect of probiotic fermented milk on (**A**) C-reactive protein [19,22,25] and (**B**) tumor necrosis factor-alpha [22,25].

The meta-analysis of three [19,22,25] showed a significant reduction in CRP (n probiotic fermented milk/n control = 64/59) by 0.25 mg/L (95% CI −0.43, −0.08 mg/L; Figure 5A) without heterogeneity (I^2^ = 0%). Two [22,25] RCTs were included in the analyses of TNF-α (n probiotic fermented milk/n control = 44/39). No significant difference in TNF-α levels was observed between probiotic and placebo users (MD: −0.58, 95% CI −1.47, 0.32, *p* = 0.21; Figure 5B), with considerable heterogeneity (I^2^ = 66%, *p* = 0.09).

## 4. Discussion

Modulating gut microbiota composition through probiotic administration has been proposed as a novel therapy for type 2 diabetes mellitus (T2DM), and fermented milk is arguably the most common and ideal probiotic carrier. In the present meta-analysis of RCTs, probiotic fermented milk supplementation the levels of fasting plasma glucose, hemoglobin A1c, TC, and CRP but did not significantly affect the levels of HOMA-IR, TG, LDL-C, HDL-C, and TNF-α.

The effects of probiotic fermented milk supplementation on glucose metabolism parameters (FPG, HbA1c, and HOMA-IR) have been previously observed in a meta-analysis of RCTs [15]. However, the present meta-analysis differs from the previous meta-analysis in some important aspects. First, although the previous and present meta-analyses included almost the same amount of RCTs (six RCTs vs. seven RCTs) for the same glucose parameters, we found that probiotic fermented milk supplementation reduced the levels of FPG and [28] HbA1c, which was different from the null effect of the supplementation on both parameters observed in the previous analysis. Second, the previous meta-analysis used the standardized mean difference (SMD) rather than WMD, which was used in the present meta-analysis. The variation in the selection of the measure of effect size could, to a certain extent, explain the discrepancy in the findings between both meta-analyses. The main difference between WMD and SMD is that the former measure of effect size is applied in meta-analysis when the studies reported having the same measurement scales and expressed in units of the measurement scales (e.g., mg/dL). In contrast, the latter measure of effect size is applied in the meta-analysis when the studies used different measurement scales and expressed in units of SD, making the overall intervention effect difficult to interpret. In our case, while not all RCTs used the same measurement scales, the scales can be easily converted to each other. Therefore, the selection of SMD in the previous meta-analysis is not justified. Third, the previous meta-analysis did not investigate the effects of probiotic fermented milk supplementation on lipid metabolism parameters and inflammatory markers, which are important factors in the progression of T2DM and its complications. Although the effects of probiotic fermented milk supplementation on lipid metabolism parameters and inflammatory markers in T2DM have not been quantified, meta-analyses of RCTs have reported the effects of probiotic fermented milk on lipid metabolism parameters [29] and inflammatory markers [28] in general participants (i.e., not restricted to T2DM). The reduced levels of TC and CRP with the supplementation and the lack of supplementation effect on the levels of LDL-C, TG, and TNF-α observed in the present meta-analysis are consistent with the previous meta-analysis in general participants.

The exact mechanisms for the beneficial effects of probiotic fermented milk on glucose and lipid metabolism and inflammation in T2DM are likely multifactorial and not fully elucidated. The most likely mechanistic explanation for such benefits is the change in the composition of the host gut microbiota towards balance after the administration of probiotics. Dysbiosis (imbalance) of gut microbiota composition is common in patients with T2DM. Dysbiosis can lead to elevated intestinal permeability, allowing bacterial endotoxins to enter the circulation, eventually leading to inflammation, insulin resistance, dyslipidemia, and hyperglycemia [9,10]. Probiotics can balance intestinal microbiota equilibrium by promoting short-chain fatty acid (SCFA)-producing bacterial growth and inhibiting the number of harmful bacteria [30,31]. The activation of G-protein-coupled receptors on L-cells by SCFAs triggers the release of glucagon-like peptide-1 and peptide YY, leading to enhanced insulin secretion, reduced glucagon secretion, and improved lipid metabolism. SCFAs can decrease intestinal permeability and circulating endotoxins, alleviating inflammation and oxidative stress [30,32,33,34].

While the present findings are encouraging, their robustness and clinical implications are hampered by several caveats. First, there was a lack of RCTs that compared probiotic fermented milk with probiotic-free control, as nearly all RCTs compared probiotic fermented milk and conventional fermented milk. This approach made it difficult to assess the true effect of probiotic fermented milk supplementation because conventional fermented milk per se contains probiotics (although in lower amounts than probiotic fermented milk). If this is truly the case, the lack of supplementation effect observed for most investigated outcomes (i.e., HOMA-IR, TG, LDL-C, HDL-C, and TNF-α) could have been due to suboptimal control selection. Second, all RCTs asked the participants in the intervention group to supplement their habitual diet with probiotic fermented milk, while those in the control group were asked to continue their habitual diet. Given that the habitual diet was not controlled in those RCTs and that diet could modify gut microbiota composition, glucose and lipid metabolism, and inflammatory response, any imbalance in dietary intake of both groups could have biased the effect of probiotic fermented milk supplementation. Based on several RCTs [21,22,23,26,27,28,29] that performed dietary intake analyses, it appeared that the difference in intake of energy and nutrient intake in both groups before and after the intervention was not significant in most cases, although the imbalance in polyunsaturated fatty acid intake before the intervention was observed [22,23]. Third, the lack of included RCTs in each analysis (even the largest analyses only included seven RCTs) reduced the robustness of the overall findings and precluded the ability to fully evaluate the potential source of heterogeneity and effect modifiers through meaningful subgroup analyses. Consequently, many important issues regarding probiotic administration, particularly the potential dose-dependent, species-specific, and strain-specific effects, could not be assessed with the current datasets. Finally, The present meta-analysis was not adequately powered to assess the potential publication bias due to the low number of included RCTs. Therefore, the potential publication bias could not be fully ruled out.

## 5. Conclusions

In summary, the present findings provide a crude indication of the potential benefits of probiotic fermented milk supplementation in improving glucose and lipid metabolism and inflammation in patients with T2DM. Unfortunately, no solid conclusions can be made based on these findings due to the considerable limitations of the included trials and analyses. More robust evidence is needed to determine the clinical significance of probiotic fermented milk in the management of T2DM.

## Figures and Tables

**Table 1:** Participants, Interventions, Comparisons, Outcomes, and Study (PICOS) design framework.

	Inclusion Criteria	Exclusion Criteria
Participants	Adult patients with type 2 diabetes mellitus patients	Patients with other types of diabetes mellitus
Intervention or exposure	Probiotics-enriched fermented milk	Conventional fermented milk
Comparison	Conventional fermented milk or non-dairy controls/placebos	
Outcome	Glucose metabolism parameters (fasting plasma glucose, hemoglobin A1c, and homeostasis model assessment of insulin resistance) Lipid metabolism parameters (total cholesterol, triglycerides, low-density lipoprotein cholesterol, and high-density lipoprotein) Inflammatory markers (tumor necrosis factor-alpha and C-reactive protein)	
Study design	Parallel or cross-over randomized controlled trial	Non-randomized study (i.e., observational study)

**Table 2:** Characteristics of the included trials.

Author Year	Country	No.of Participants in the Probiotics Group/No. of Participants in the Placebo Group	Age in Intervention Group (mean ± SD)	Age in Control Group (mean ± SD)	Treatment (Bacteria)	Control (Bacteria)	Duration (Weeks)	Outcomes
Ejtahed(a). 2011 [21]	Iran	30/30	50.87 ± 1.40	51.00 ± 1.34	300 g/d probiotic-enriched yogurt (original culture: *Lactobacillus bulgaricus* and *Streptococcus thermophilus*; additional culture (3.98 × 10^9^ cfu/d): *Lactobacillus acidophilus* La5 (7.23 × 10^6^ cfu/g on day 1) and *Bifidobacterium lactis* Bb12 (6.04 × 10^6^ cfu/g on day 1))	300 g/d conventional yogurt (original culture: *Lactobacillus bulgaricus* and *Streptococcus thermophilus*)	6	TC, TG, HDL-C, LDC-C
Ejtahed(b). 2012 [20]	Iran	30/30	50.87 ± 7.68	51.00 ± 7.32	300 g/d of probiotic-enriched yogurt (original culture: *Lactobacillus bulgaricus* and *Streptococcus thermophilus*; additional culture (3.98 × 10^9^ cfu/d): *Lactobacillus acidophilus* La5 (7.23 × 10^6^ cfu/g on day 1) and *Bifidobacterium lactis* Bb12 (6.04 × 10^6^ cfu/g on day 1))	300 g/d of conventional yogurt (original culture: *Lactobacillus bulgaricus* and *Streptococcus thermophilus*)	6	FPG, HbA1c
Mohamadshahi(a), 2014 [24]	Iran	22/22	51	51	300 g/d probiotic-enriched yogurt (original culture: *Lactobacillus bulgaricus* and *Streptococcus thermophilus*; additional culture (2.22 × 10^9^ cfu/d): *Lactobacillus acidophilus* La5 (3.7 × 10^6^ cfu/d on day 1) and *Bifidobacterium lactis* Bb12 (3.7 × 10^6^ cfu/d on day 1))	300g/d of conventional yogurt (original culture: *Lactobacillus bulgaricus* and *Streptococcus thermophilus*)	8	TC, TG, HDL-C, LDL-C
Mohamadshahi(b), 2014 [25]	Iran	22/21	53.00 ± 5.9	49.00 ± 7.08	Probiotic-enriched yogurt (original culture: *Lactobacillus bulgaricus* and *Streptococcus thermophilus*; additional culture (2.22 × 10^9^ cfu/d): *Lactobacillus acidophilus* La5 (3.7 × 10^6^ cfu/d on day 1) and *Bifidobacterium lactis* Bb12 (3.7 × 10^6^ cfu/d on day 1))	Conventional yogurt (original culture: *Lactobacillus bulgaricus* and *Streptococcus thermophilus*)	8	FPG, HbA1c, CRP, TNF-α, IL-6
Hove, 2015 [22]	Denmark	23/18	58.5 ± 7.7	60.6 ± 5.2	300 g/d of Commercial probiotic yogurt ‘Cardi04’ (*Lactobacillus helveticus* Cardi04)	300 g/d of probiotics-free acidified milk	12	FPG, HbA1c, HOMA-IR, TC, TG, HDL-C, LDL-C, CRP, TNF-α
Ostadrahimi, 2015 [26]	Iran	30/30	no	no	600 g/d of probiotic-enriched kefir (original cultures: *Streptococcus thermophilus*; additional cultures (2.88 × 10^10^ cfu/d): *Lactobacillus casei* (1.5 × 10^7^ cfu/g on day 1), *Lactobacillus* *acidophilus* (2.5 × 10^7^ cfu/g on day 1), and *bifidobacterium lactis* (8 × 10^6^ cfu/g on day 1))	600 g/d of conventional fermented milk (original cultures: *Streptococcus thermophilus*)	8	FPG, HbA1c, TC, TG, HDL-C, LDL-C,
Bayat, 2016 [19]	Iran	20/20	54.1 ± 9.54	46.95 ± 9.34	150 g/d of probiotic yogurt (not reported)	Dietary advice (not applicable)	8	FPG, HbA1c, TC, TG, LDL-C, HDL-C, CRP
Alihosseini, 2017 [18]	Iran	30/30	Not reported	Not reported	600 g/d of probiotic-enriched kefir (original cultures: *Streptococcus thermophilus*; additional cultures (2.88 × 10^10^ cfu/d): *Lactobacillus casei* (1.5 × 10^7^ cfu/g on day 1), *Lactobacillus* *acidophilus* La5 (2.5 × 10^7^ cfu/g on day 1), and *bifidobacterium lactis* Bb12 (8 × 10^6^ cfu/g on day 1))	600 g/d of conventional fermented milk (original cultures: *Lactobacillus bulgaricus* and *Streptococcus thermophilus*)	8	HOMA-IR
Tonucci, 2017 [27]	Brazil	23/22	51.83 ± 6.64	50.95 ± 7.20	120 g/d of probiotic fermented goat milk (original culture (6.26 × 10^10^ cfu/d): *Streptococcus thermophilus*; *Lactobacillus acidophilus* La5 (7.72 × 10^7^ cfu/g on day 1) and *Bifidobacterium lactis* Bb12 (4.45 × 10^8^ cfu/g on day 1))	120 g/d of conventional fermented goat milk (original culture: *Streptococcus thermophilus*)	6	FPG, HbA1c, HOMA-IR, TC, HDL-C, LDL-C
Mirjalili, 2023 [23]	Iran	36/36	54.5 ± 8.0	58.1 ± 9.8	100 g/d of probiotic-enriched yogurt (original culture: *Lactobacillus bulgaricus* and *Streptococcus thermophilus*; additional culture (7.3 × 10^8^ cfu/d): *Lactobacillus acidophilus* La5 and *Bifidobacterium lactis* Bb12 (7.3 × 10^6^ cfu/d on day 1))	100 g/d of conventional yogurt (original culture: *Lactobacillus bulgaricus* and *Streptococcus thermophilus*)	12	FPG, HbA1c, TC, TG, HDL-C, LDL-C

## Data Availability

Not applicable.

## Supplementary Materials

The following supporting information can be downloaded at: https://www.mdpi.com/article/10.3390/biology13080641/s1, Figure S1: Forest plot for the effect of probiotic yogurt on Body Mass Index; Figure S2: Forest plot for the effect of probiotics on HOMA-IR compared to controls in pooled analysis; Figure S3: Forest plot for the effect of probiotics on HDL compared to controls in pooled analysis; Figure S4: Forest plot for the effect of probiotics on CRP compared to controls in pooled analysis; Table S1: Full detail of the search strategy.
