# Supplementary material for: Effect of Probiotic Fermented Milk Supplementation on Glucose and Lipid Metabolism Parameters and Inflammatory Markers in Patients with Type 2 Diabetes Mellitus: A Meta-Analysis of Randomized Controlled Trials
Source: Biology (Basel). 2024 Aug 21;13(8):641. doi: 10.3390/biology13080641 (PMC11351427; doi:10.3390/biology13080641)
Supplement: Supplementary file 1 [file biology-13-00641-s001.zip › biology-3133993-supplementary.pdf]

Table S1 Full details of the search strategy

Database	Step	Search Syntax	Total retrieves
PubMed	1	Search ("Diabetes Mellitus, Type 2"[Mesh]) OR (Diabetes Mellitus, Type 2[Title/Abstract]) OR (Type 2 diabetes[Title/Abstract]) OR (Diabetes Mellitus[Title/Abstract]) OR (Type 2 Diabetes Mellitus[Title/Abstract]) OR (Diabetes, Type 2[Title/Abstract])	471998
	2	Search ("Probiotics"[Mesh]) OR (Probiotic[Title/Abstract]) OR (Probiotic fermented milk[Title/Abstract]) OR (Probiotic yogurt[Title/Abstract]) OR (Fermented Food[Title/Abstract]) OR (Fermented Milk[Title/Abstract])) OR (Fermented Dairy[Title/Abstract]) OR (Cultured Milk[Title/Abstract]) OR (Sour Milk[Title/Abstract]) OR (Buttermilk[Title/Abstract]) OR (Yogurt[Title/Abstract]) OR (Yoghurt[Title/Abstract]) OR (Fermented dairy foods[Title/Abstract])) OR (fermented dairy products[Title/Abstract]) OR (fermented milk products[Title/Abstract]) OR (fermented milk foods[Title/Abstract]) OR (kefir[Title/Abstract]) OR (kefir products[Title/Abstract])	45084
	3	Search ("Inflammation"[Mesh]) OR (Inflammation[Title/Abstract]) OR (Inflammation factor[Title/Abstract]) OR (Inflammatory[Title/Abstract]) OR (inflammatory marker[Title/Abstract]) OR (Interleukin[Title/Abstract]) OR (TNF-α[Title/Abstract]) OR (IL-6[Title/Abstract])) OR (IL-8[Title/Abstract]) OR (IL-1β[Title/Abstract]) OR (IL-6[Title/Abstract]) OR (Tumor factor[Title/Abstract])	1680658
	4	1 AND 2 AND 3	528
Web of Science	1	(TS=(Diabetes Mellitus, Type 2) OR AB=(Diabetes Mellitus, Type 2 OR Type 2 diabetes OR Diabetes Mellitus OR Type 2 Diabetes Mellitus OR Diabetes, Type 2)) AND (TS=(Probiotic) OR AB=(Probiotic OR Probiotic fermented milk OR Probiotic yogurt OR Fermented Food OR Fermented Milk OR Fermented Dairy OR Cultured Milk OR Sour Milk OR Buttermilk OR Yogurt OR Yoghurt OR Cheese OR Fermented dairy foods OR fermented dairy products OR fermented milk products OR fermented milk foods OR kefir OR kefir products)) AND (TS=(Inflammation) OR AB=(Inflammation OR Inflammation factor OR Inflammatory OR inflammatory marker OR TNF-α OR IL-6 OR IL-8 OR IL-1β OR Tumor factor))	895

Table S1 (continued)

Database	Step	Search Syntax	Total retrieves
Cochrane library	1	Search (Diabetes Mellitus, Type 2 or Type 2 diabetes or Diabetes Mellitus or Insulin resistance or Type 2 Diabetes Mellitus or Diabetes, Type 2)	101596
	2	Search (Probiotic OR Probiotic fermented milk OR Probiotic yogurt OR Fermented Food OR Fermented Milk OR Fermented Dairy OR Cultured Milk OR Sour Milk OR Buttermilk OR Yogurt OR Yoghurt OR Fermented dairy foods OR fermented dairy products OR fermented milk products OR fermented milk foods OR kefir OR kefir products)	13103
	2	Search (Inflammation OR Inflammation factor OR Inflammatory OR inflammatory marker OR Interleukin OR TNF-α OR IL-6 OR IL-8 OR IL-1β OR Tumor factor))	142439
	4	#1 and #2 and #3	361

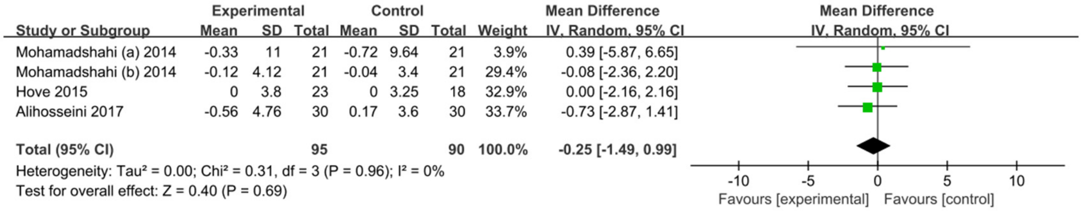

Figure S1. Forest plot of the effect of probiotic yogurt on Body Mass Index.

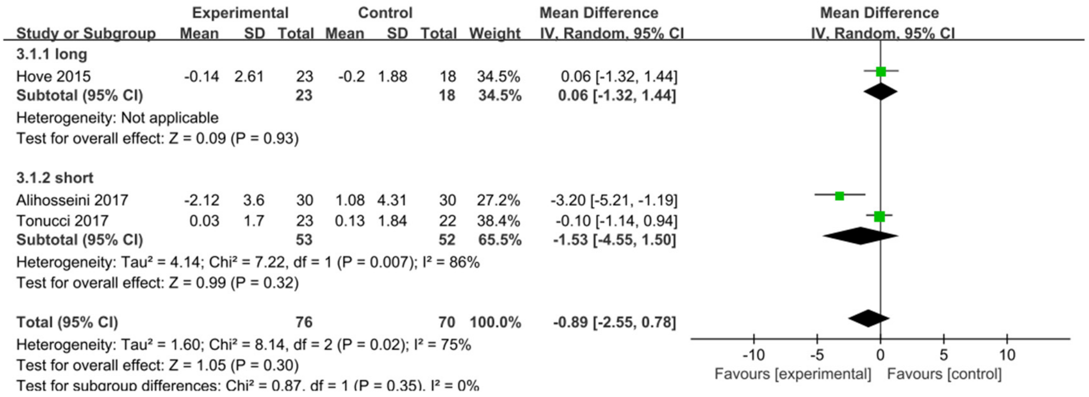

Figure S2. Forest plot of the effect of probiotics on HOMA-IR compared to controls in

pooled analysis. The shaded diamonds indicate the effect of probiotics in a particular study (weighted difference in mean). The horizontal lines represent 95% confidence intervals (CIs). The figure shows the summary of studies overall and subdivided by length of intervention. “long”: 12 weeks or longer; “short”: 8 weeks or shorter.

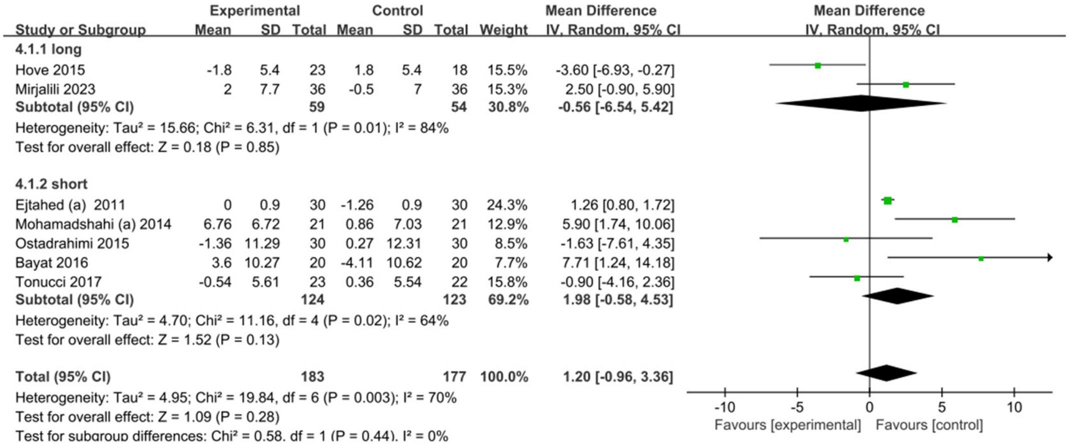

Figure S3. Forest plot of the effect of probiotics on HDL compared to controls in pooled analysis. The figure shows the summary of studies overall and subdivided by length of intervention. “long”: 12 weeks or longer; “short”: 8 weeks or shorter.

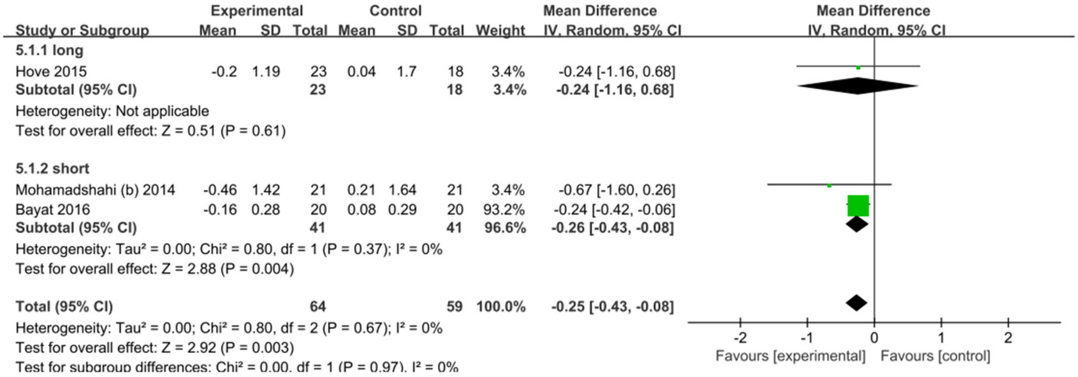

Figure S4. Forest of for the effect of probiotics on CRP compared to controls in pooled analysis. The figure shows the summary of studies overall and subdivided by length of intervention. “long”: 12 weeks or longer; “short”: 8 weeks or shorter.
